# New estimates of the Zika virus epidemic attack rate in Northeastern Brazil from 2015 to 2016: A modelling analysis based on Guillain-Barré Syndrome (GBS) surveillance data

**DOI:** 10.1371/journal.pntd.0007502

**Published:** 2020-04-29

**Authors:** Daihai He, Shi Zhao, Qianying Lin, Salihu S. Musa, Lewi Stone

**Affiliations:** 1 Department of Applied Mathematics, Hong Kong Polytechnic University, Hong Kong, China; 2 Division of Biostatistics, JC School of Public Health and Primary Care, Chinese University of Hong Kong, Hong Kong, China; 3 Clinical Trials and Biostatistics Lab, Shenzhen Research Institute, Chinese University of Hong Kong, Shenzhen, China; 4 Michigan Institute for Data Science, University of Michigan, Ann Arbor, Michigan, United States of America; 5 Mathematical Science, School of Science, RMIT University, Melbourne, Victoria, Australia; 6 Biomathematics Unit, School of Zoology, Faculty of Life Sciences, Tel Aviv University, Tel Aviv, Israel; University of California, Davis, UNITED STATES

## Abstract

**Background:**

Between January 2015 and August 2016, two epidemic waves of Zika virus (ZIKV) disease swept the Northeastern (NE) region of Brazil. As a result, two waves of Guillain-Barré Syndrome (GBS) were observed concurrently. The mandatory reporting of ZIKV disease began region-wide in February 2016, and it is believed that ZIKV cases were significantly under-reported before that. The changing reporting rate has made it difficult to estimate the ZIKV infection attack rate, and studies in the literature vary widely from 17% to > 50%. The same applies to other key epidemiological parameters. In contrast, the diagnosis and reporting of GBS cases were reasonably reliable given the severity and easy recognition of the disease symptoms. In this paper, we aim to estimate the real number of ZIKV cases (i.e., the infection attack rate) and their dynamics in time, by scaling up from GBS surveillance data in NE Brazil.

**Methodology:**

A mathematical compartmental model is constructed that makes it possible to infer the true epidemic dynamics of ZIKV cases based on surveillance data of excess GBS cases. The model includes the possibility that asymptomatic ZIKV cases are infectious. The model is fitted to the GBS surveillance data and the key epidemiological parameters are inferred by using a plug-and-play likelihood-based estimation. We make use of regional weather data to determine possible climate-driven impacts on the reproductive number $R_{0}$, and to infer the true ZIKV epidemic dynamics.

**Findings and conclusions:**

The GBS surveillance data can be used to study ZIKV epidemics and may be appropriate when ZIKV reporting rates are not well understood. The overall infection attack rate (IAR) of ZIKV is estimated to be 24.1% (95% confidence interval: 17.1%—29.3%) of the population. By examining various asymptomatic scenarios, the IAR is likely to be lower than 33% over the two ZIKV waves. The risk rate from symptomatic ZIKV infection to develop GBS was estimated as *ρ* = 0.0061% (95% CI: 0.0050%—0.0086%) which is significantly less than current estimates. We found a positive association between local temperature and the basic reproduction number, $R_{0}$. Our analysis revealed that asymptomatic infections affect the estimation of ZIKV epidemics and need to also be carefully considered in related modelling studies. According to the estimated effective reproduction number and population wide susceptibility, we comment that a ZIKV outbreak would be unlikely in NE Brazil in the near future.

## Author summary

The mandatory reporting of the Zika virus (ZIKV) disease began region-wide in February 2016, and it is believed that ZIKV cases could have been highly under-reported before that. Given the Guillain-Barré syndrome (GBS) is relatively well reported, the GBS surveillance data has the potential to act as a reasonably reliable proxy for inferring the true ZIKV epidemics. We developed a mathematical model incorporating weather effects to study the ZIKV-GBS epidemics and estimated the key epidemiological parameters. It was found that the attack rate of ZIKV was likely to be lower than 33% over the two epidemic waves. The risk rate from symptomatic ZIKV case to develop GBS was estimated to be approximately 0.0061%. The analysis suggests that it would be difficult for another ZIKV outbreak to appear in Northeastern Brazil in the near future.

## Introduction

The Zika virus (ZIKV) was first identified in 1947 in the Zika forest of Uganda [1], and within a few years was found spreading in human populations of Nigeria [2, 3]. Transmitted through the bites of mosquito vectors (usually of the *Aedes* genus), ZIKV is an arbovirus from the family *Flaviviridae* [4, 5]. Other transmission routes have also been found (materno-fetal, sexual transmission, and via blood transfusion) but they are less common [6, 7, 8, 9]. By the 1970s, the virus was circulating widely in West Africa, although it was considered a relatively mild human infection that generally results in only fever, rash and possibly conjunctivitis [3, 10]. By 2007, the virus had escaped Africa to the island of Yap in Micronesia where, according to some estimates, it infected up to 75% of the island population [11]. ZIKV reached Polynesia in 2013, and at least by 2015, it had invaded Brazil and then very quickly the rest of South America where it reached epidemic levels [12, 13].

Since its appearance in French Polynesia and Brazil, the virus has been associated with severe neurological disorders linked to birth defects. ZIKV infection was found to pass from mother to fetus during pregnancy with the potential to result in microcephaly which causes fetal abnormalities including possible skull collapse [5]. In addition, since 2014 ZIKV was found to be strongly associated with the Guillain-Barré syndrome (GBS) amongst a small proportion of those infected [14, 15]. GBS can result in long-term muscle weakness, pain, and in some circumstances death [16]. Many studies found a causal link between GBS and ZIKV disease [17, 18, 19]. In summary, GBS has many times been associated with ZIKV outbreaks in many countries [15], making the empirical association unusually strong.

**Fig 1:**
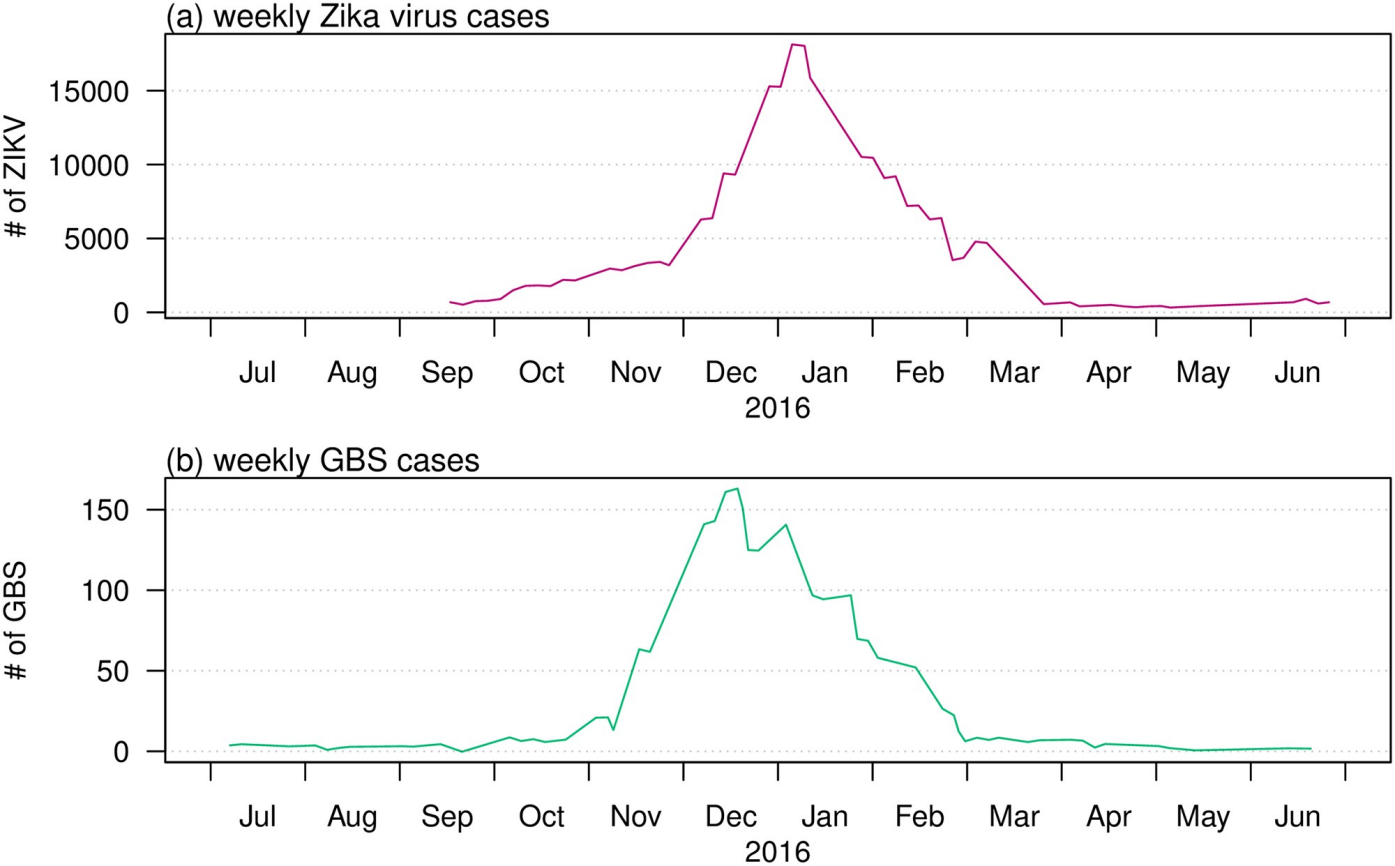
Total ZIKV (top) and GBS (bottom) cases as time series summed over the different states and countries: Bahia State, Colombia, the Dominican Republic, El Salvador, Honduras, Suriname, and Venezuela from April 01 of 2015 to March 31 of 2016. Data from Ref. [25]. Note that the data shown in this figure were merely to illustrate the match between ZIKV and GBS cases data, and the data are not involved in the modelling analysis.

While considered relatively benign for decades since 1947, ZIKV disease suddenly became a major global disease threat. A Public Health Emergency of International Concern (PHEIC) was announced by the WHO on February 01, 2016 [20], in the lead-up to the Rio Olympic Games in Brazil. But until then, because of the relatively low interest in the ZIKV, surveillance in most areas was of low quality with poor coverage and consequently a large under-reporting of cases. There was little knowledge of key parameters: for example the true attack rate, the proportion of asymptomatic cases amongst infected ZIKV cases, the reproductive number. This has led to stepped up activity in surveillance and modelling efforts in recent years. But given the poor case-data available and the lack of knowledge of a reporting rate (which changed significantly in time and location) for those infected with ZIKV, results from modelling efforts have often proved to be inconsistent. Here, we take a new approach that attempts to overcome some of the problems associated with the large uncertainties associated with the reporting of ZIKV cases. Instead, we work with time series of GBS cases which should be far more reliable. We argue that a high proportion of people infected with GBS will in fact report to the doctor. Fig 1 makes clear the strong association between ZIKV cases and GBS by plotting reported cases of both diseases on the same axes. It is clear that the dynamics of the two diseases are closely in step. The unique feature of our work is that we draw on this property and fit our model to GBS data collected during and following the period of a ZIKV outbreak. We use this to infer the true numbers, and dynamics in time, of ZIKV cases.

For the modelling work that follows, it is useful to consider some of the above events in more detail on a country-specific basis. This provides further important background information that justifies our approach in using GBS as a proxy for Zika-cases, on data sources and on choices of parameter values.

### French Polynesia

From October 2013 to April 2014, a severe ZIKV outbreak hit French Polynesia, and the attack rate (IAR) was first estimated as 66% [21], but updated soon after to 49% [22]. An outbreak of 42 GBS cases was simultaneously reported, but with a three-week delay in the peak timing, and was linked to the ZIKV outbreak [23]. Based on the IAR of [22], the risk of ZIKV induced GBS can thus be roughly calculated as 0.32 GBS cases per 1,000 ZIKV infections, or just *ρ* = 0.00032. [23] estimated the proportion to be *ρ* = 0.00024. Aubry *et al*. also found that, the ratio of asymptomatic to symptomatic infections (asymptomatic ratio) was about 1:1 in the general population and 1:2 among school children [22]. These findings are notably different from estimates for a previous ZIKV outbreak in Yap island in 2007, where the asymptomatic ratio was 4.4:1 and the estimated overall ZIKV IAR was about 75% [11].

Following the ZIKV outbreak in French Polynesia, the region experienced a Chikungunya virus (CHIKV) disease outbreak with an estimated 66,000 cases from October 2014 to March 2015, and 9 GBS cases occurred [24]. The crude risk of CHIKV induced GBS was found to be 0.136 per 1,000 CHIKV infections. Thus, based on these studies [23, 24], a ZIKV infection is of (0.32 ÷ 0.136 =) 2.35-fold more likely to induce GBS when compared to a CHIKV infection. Cauchemez *et al*. (2016) [21] also found that the risk of ZIKV induced microcephaly was 95 cases (34-191) per 10,000 women infected in their first trimester during 2013-14.

### Northeastern Brazil

**Fig 2:**
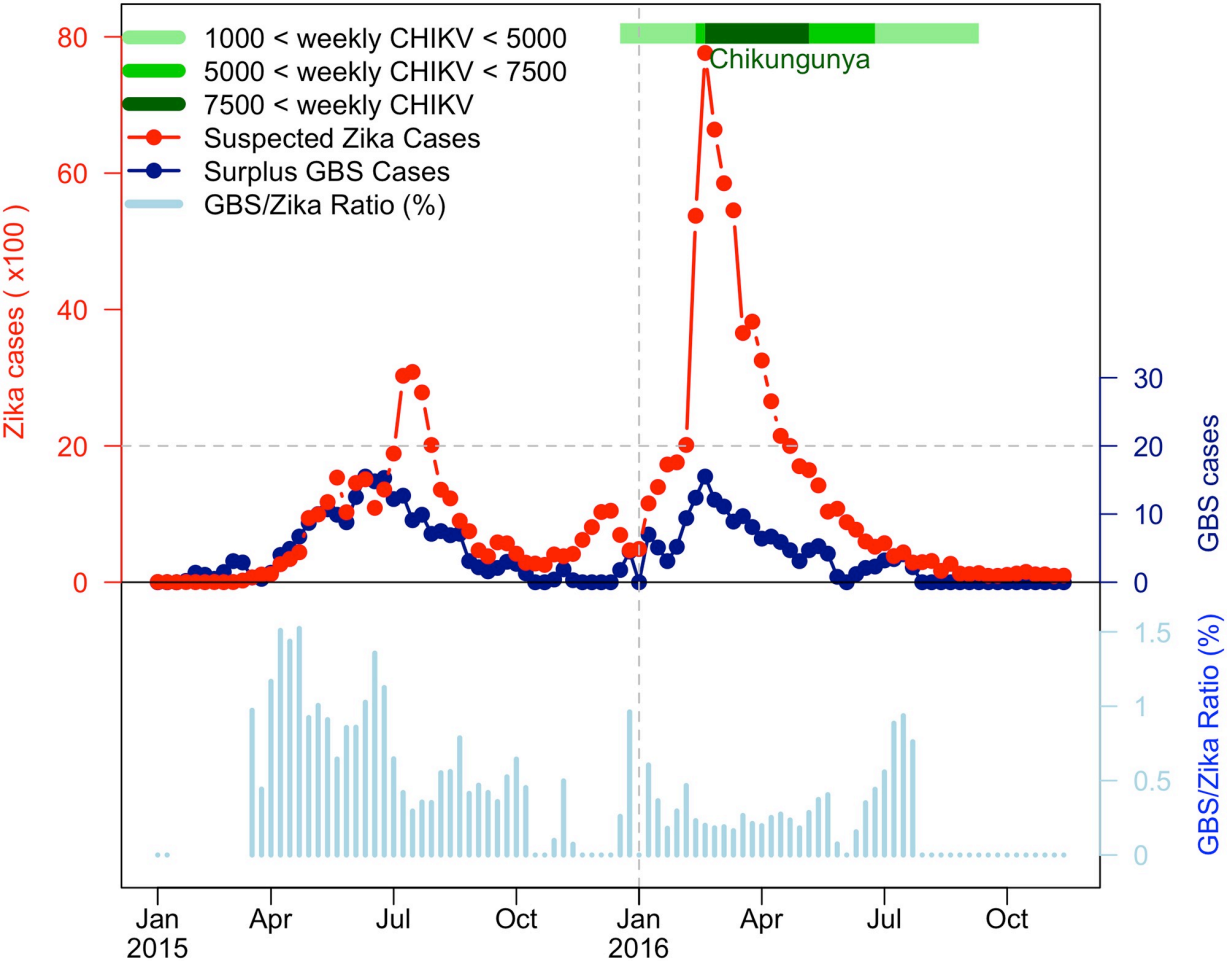
The reported ZIKV cases, excess (or surplus) GBS cases and GBS-to-ZIKV ratio in the NE region of Brazil from January 2015 to November 2016. The red dotted line represents weekly ZIKV disease cases, the dark blue dotted line represents weekly surplus GBS cases and the light blue bars are GBS-to-ZIKV ratios. The CHIKV outbreak of 2016 is denoted with different shades of green according to number of weekly cases. The GBS-to-ZIKV ratios are not plotted for the initial few weeks as the scale of the ZIKV data is not large enough to compute a meaningful ratio.

The Northeastern (NE) region of Brazil was the hardest-hit region in the Americas during 2015-16. In this period three mosquito-borne diseases—dengue virus, ZIKV, and CHIKV, co-circulated, and weekly cases were documented [26]. In addition, local GBS and microcephaly cases were also recorded. Over the two years, two waves of ZIKV disease were accompanied by two waves of reported GBS cases, as shown in Fig 2, which indicated a possible epidemiological association. A striking wave of microcephaly cases with a 23-week delay to the first ZIKV wave was identified and discussed in [26]. The delay arises because ZIKV infections in the first trimester of pregnancy are most likely to induce microcephaly [21, 27, 28, 29]).

A substantial CHIKV wave was also observed during the second ZIKV wave in 2016 as indicated in Fig 2 and [26]. CHIKV can induce GBS with a smaller risk ratio (1 to 2.35) than ZIKV as discussed above and according to results in [24, 30, 31, 32, 33]. Note that in the latter studies, no cases of GBS induced by dengue epidemics were reported. One recent cohort study was conducted on 345 pregnant women with ZIKV rash observed (presenting at the Oswaldo Cruz Foundation) in Rio de Janeiro (the largest city in Eastern Brazil) between September 2015 and May 2016 [29]. The IAR of CHIKV was found to be approximately 17%; and in contrast, the IAR of ZIKV was 53%, as based on PCR tests. In addition, a strong cross-protection between ZIKV and CHIKV was also observed, but no cross-protection was observed between ZIKV and dengue virus (DENV). The IAR of CHIKV was 21.1%, and 41.7% for ZIKV-negative women while only 2.8% of ZIKV-positive women were infected with CHIKV. Thus, among pregnant women with rash observed in this period, the ratio of ZIKV and CHIKV is (roughly) 5 to 2. Evident cross-protection between CHIKV and ZIKV (but not between DENV and ZIKV) can be deduced from the same study with the same women [29]. Therefore, we suspect that the two waves of excess GBS cases in NE Brazil were largely due to ZIKV disease rather than CHIKV, for two reasons: (i) ZIKV is 2.35-fold likely to induce GBS than CHIKV; and (ii) ZIKV IAR could be three times higher than that of CHIKV based on the Rio de Janeiro study [29] to project the situation in NE Brazil.

Our work is based on the fact that it is difficult to estimate the infection attack rate (IAR) of ZIKV directly from the reported ZIKV cases time series given the non-constant reporting efforts over 2015 and 2016. In the literature, estimates of the IAR of ZIKV in Brazil (especially Northeast Region of Brazil) vary from less than 20% to more than 60%, and thus appear inconclusive. A summary table is provided in the S1 Text. Most previous works were based on unreliable ZIKV surveillance data. In this work, we aim to use the relatively reliable GBS data in NE Brazil to infer the ZIKV epidemic.

The under-reporting of ZIKV cases in 2015 also appears to be reflected in what was felt to be a high number of microcephaly cases (after a 26-week delay [26]). This is because microcephaly cases are easier to identify and are thus better reported [20]. Nevertheless, the reporting criteria of microcephaly cases also changed significantly over the two years [34] leading to overall unreliable estimates. Given this known and documented unreliability [34], we felt it might not be wise to estimate IAR of ZIKV directly based on the reported number of microcephaly cases. Also, we would need to consider the effect of time-varying birth rates if using microcephaly cases to infer ZIKV cases.

However, it seems a reasonable approximation to assume that the number of GBS cases per ZIKV infected individual should remain constant in time, and that the reported GBS cases are relatively well reported over time. The reporting criteria of GBS should be relatively accurate and stable owing to the distinct identifiable and severe clinical features of GBS [16]. By assuming the GBS-ZIKV risk ratio is constant, we attempted to fit an epidemic model and infer this ratio based on the GBS cases time series. Because of the co-circulation of both dengue fever and ZIKV during the two waves, misdiagnoses of ZIKV could occur [29, 27, 26], especially given both diseases have similar symptoms. Nevertheless, no GBS induced DENV was reported in the 2015 and 2016 years. Thus, the large-scale ZIKV outbreak was the major source of the excess GBS cases [26]. For these reasons, we use the excess GBS cases time series to infer the pattern of ZIKV outbreak and the overall IAR of ZIKV in Northeastern Brazil.

Mathematical modelling provides a possible way to infer the epidemic waves of ZIKV (or together with a minor proportion of CHIKV). First, we assume a constant risk ratio between symptomatic ZIKV cases and reported GBS cases (ZIKV-GBS ratio), denoted by *ρ*. Second, we simulate our ZIKV model, and fit the model to observed GBS cases with a time-dependent ZIKV transmission rate. Finally, by using iterated filtering techniques, we find the maximum likelihood estimates of *ρ* and the overall IAR.

## Data and methods

### Data

Brazil has a territory of more than 8.5 million km^2^ and is one of the largest countries in the world with an estimated population of 211 million [35]. The Northeastern (NE) region of Brazil is one of five regions, and has an area of 1.6 million km^2^. The region has a population of 57 million, but 75% of the population live in the major cities of the coastal area which stops at the Atlantic on the East. While larger Brazil exhibits spatially heterogeneous climate trends, a large part of the heterogeneity can be attributed to its five relatively homogeneous climate regions [36]. We are interested in the NE Brazil climate region, which temporally has a known seasonal cycle in terms of climate variables, e.g., temperature and rainfall, that impresses itself over all the region, and is relatively spatially uniform especially over the coastal areas.

The reported weekly excess (or surplus) GBS cases time series of NE Brazil, from Jan 2015 to Nov 2016, were kindly provided by Professor Oliveira from the Ministry of Health in Brazil, as used in their important recent study [26]. The time series are plotted in Fig 2 with datasets of ZIKV and CHIKV for the period. The GBS data used in this work follow the case definitions given in S1 Text. In Fig 2 we observe that the GBS-to-ZIKV ratio of 2016 was significantly lower than in 2015, which was likely due to the under-reporting of ZIKV epidemic before 2016 [20].

Daily mean temperature and total rainfall (beginning from December 1, 2014) data were obtained from six cities having some of the largest populations in NE Brazil (source: https://www.worldweatheronline.com/). A map of the locations of the six cities is given in the S1 Text. We calculated the daily average temperature and the total rainfall across the six cities and smoothed them with R function *smooth*, and used these averages to model the overall situation in the large area as NE Brazil [37, 38, 39]. Spatially speaking, the relative climate homogeneity is evident from studying the data for coastal cities [36], as displayed in the figures in S1 Text. We justify our methodology of spatially aggregating the climate data in S1 Text. We provide the GBS surveillance data and weather data in the S1 Text.

### Methods

In previous work [6, 40], we developed a ZIKV transmission model, including both host and vector, based on mosquito-borne and sexual (human-to-human) transmission of ZIKV. Hosts infected with ZIKV generate a proportion of GBS cases as determined by *ρ* which is the ratio of reported GBS cases to symptomatic ZIKV cases. In our earlier work, asymptomatic ZIKV cases were assumed to be non-infectious. However, in this work the asymptomatic ZIKV cases are now assumed to be infectious, and we study their impact on the estimation of IAR and the ratio (*ρ*). The basic reproduction number ($R_{0}$) of the model is derived and estimated. We apply the plug-and-play likelihood-based inference framework for model fitting [41].

#### ZIKV-GBS model

**Fig 3:**
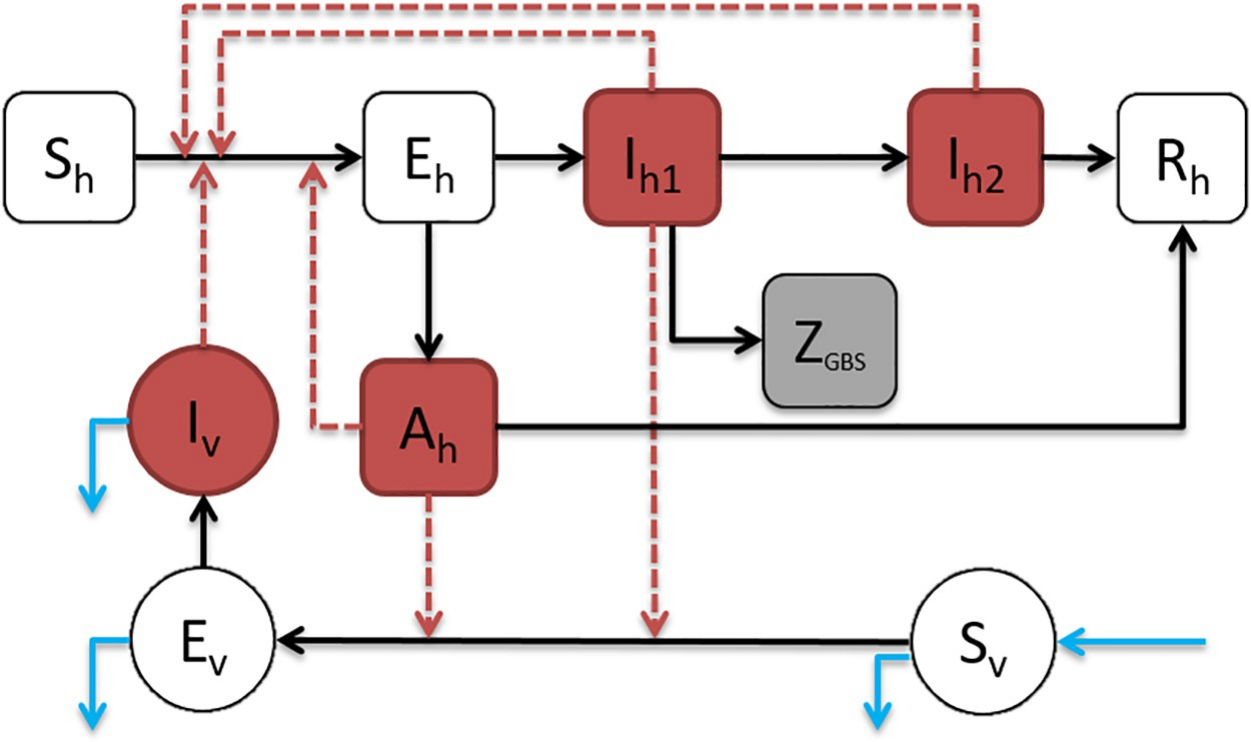
Diagram of the ZIKV-GBS epidemic model. The black arrows represent the infection status transition paths. Red dashed arrows represent transmission paths, and the light blue arrows represent the natural birth and death of mosquito vectors. Square compartments represent the host classes, and circular compartments represent the vector classes. Red compartments represent infectious classes, and the grey compartment is the (weekly) excess GBS cases (*Z*_GBS_). *S*_*h*_, *E*_*h*_, *I*_*h*_, *R*_*h*_ represents the numbers of Susceptible, Exposed, Infected and Recovered host population with respect to ZIKV, respectively. Please see text below Eq (1) for complete listing of all compartment codes.

Fig 3 shows the model diagram of the ZIKV disease transmission pathways of both humans and the mosquito vector. Following our previous work [6, 40], we continue to assume that hosts infected with ZIKV are infectious during the convalescent stage and can infect other susceptible hosts through sexual transmission [8, 9]. However, they are assumed to be noninfectious to susceptible mosquito vectors [22, 42, 43].

It is supposed that the asymptomatic cases are infectious at a weaker level than symptomatic cases and do not develop to the convalescent stage, which is biologically and clinically reasonable [8, 9]. We therefore arrive at the following ordinary differential equation (ODE) system (1).

$$\begin{array}{c} \left\{ \begin{array}{c} \begin{array}{cc} S_{h}^{\prime } & =-ab\cdot \frac{I_{v}}{N_{h}}S_{h}-\beta \cdot \frac{\eta A_{h}+I_{h1}+\tau I_{h2}}{N_{h}}S_{h},\\ E_{h}^{\prime } & =(ab\cdot \frac{I_{v}}{N_{h}}+\beta \cdot \frac{\eta A_{h}+I_{h1}+\tau I_{h1}}{N_{h}})S_{h}-\sigma_{h}E_{h},\\ A_{h}^{\prime } & =\left(1-\theta \right)\cdot \sigma_{h}E_{h}-\gamma_{h}A_{h},\\ I_{h1}^{\prime } & =\theta \cdot \sigma_{h}E_{h}-\gamma_{h1}I_{h1},\\ I_{h2}^{\prime } & =\gamma_{h1}I_{h1}-\gamma_{h2}I_{h2},\\ R_{h}^{\prime } & =\gamma_{h}A_{h}+\gamma_{h2}I_{h2},\\ Z_{\text{GBS}}^{\left(i\right)} & =\int_{\text{week}\;i}\rho \gamma_{h1}I_{h1}\;\text{d}t,\\ S_{v}^{\prime } & =B_{v}\left(t\right)-ac\cdot \frac{\eta A_{h}+I_{h1}}{N_{h}}S_{v}-\mu_{v}S_{v},\\ E_{v}^{\prime } & =ac\cdot \frac{\eta A_{h}+I_{h1}}{N_{h}}S_{v}-\left(\sigma_{v}+\mu_{v}\right)E_{v},\\ I_{v}^{\prime } & =\sigma_{v}E_{v}-\mu_{v}I_{v}. \end{array} \end{array}\right. \end{array}$$ (1)

Here, *S*_*h*_ is the susceptible host class, *E*_*h*_ is the exposed host class (i.e., within ZIKV infection latent period), *A*_*h*_ denotes the asymptomatic host class, *I*_*h*1_ denotes the host class infected with ZIKV, *I*_*h*2_ denotes the convalescent host class, and *R*_*h*_ denotes the host’s recovered class. The variable $Z_{\text{GBS}}^{\left(i\right)}$ denotes the simulated weekly excess (or surplus) reported GBS cases for the *i*-th week during the study period. *S*_*v*_ is the susceptible vector class, *E*_*v*_ is the exposed vector (i.e., within ZIKV infection latent period) and *I*_*v*_ denotes the infectious vector class. The parameter *ρ* denotes the ratio of reported (excess) GBS cases per symptomatic case of ZIKV. The model (1) parameters are summarised in Table 1.

**Table 1:** Summary table of model parameters in Eq (1). The “H” denotes human hosts’ population, and “V” denotes mosquito vectors’ population. “X→Y” denotes ZIKV infected class X infects the (ZIKV) susceptible class Y.

Parameter	Notation	(Value)/Range	Remark/Unit	Status	Source(s)
Mosquito biting rate	*a*	(0.5) 0.3—1.0	per vector⋅day	fixed	[6, 44, 45]
Transmission prob. of host	*b*	(0.4) 0.10—0.75	per bite	fixed	[6, 44, 45]
Transmission prob. of vector	*c*	(0.5) 0.30—0.75	per bite	fixed	[46]
Transmission rate by contact	*β*	(0.05) 0.001—0.10	per day	fixed	[6]
Host latent period	$\sigma_{h}^{-1}$	(5) 2—7	days	fixed	[10, 47]
Vector latent period	$\sigma_{v}^{-1}$	(10) 8—12	days	fixed	[45, 48]
Asymptomatic infectious period	$\gamma_{h}^{-1}$	(7) 5—10	days	assumed	Nil
Infectious period	$\gamma_{h1}^{-1}$	(5) 3—7	days	fixed	[6, 47]
Convalescent infectious period	$\gamma_{h2}^{-1}$	(25) 14—30	days	fixed	[42, 43]
Proportion of symptomatic	*θ*	(50%) 20%—80%	Nil	to be estimated	[22]
Infectivity scale of asymptomatic	*η*	0.0—0.99	H→H, H→V	to be estimated	Nil
Infectivity scale of convalescent	*τ*	(0.3) 0.01—0.99	H→H	fixed	[6]
Female vector lifespan	$\mu_{v}^{-1}$	(14) 4—35	days	fixed	[49, 50, 51]
Ratio: $\frac{\text{reported}\;\text{GBS}}{\text{symptomatic}\;\text{ZIKV}}$	*ρ*	0.001%—0.1%	Nil	to be estimated	[15, 22, 23]
Ratio: $\frac{\text{mosquito}\;\text{population}}{\text{human}\;\text{population}}$	*m*(*t*)	0—20	time-dependent	to be estimated	[6, 40, 44]
Initial susceptible proportion	*S*_*h*_(0)/*N*_*h*_	0.25—1.0	Nil	to be estimated	[6]

In addition,

$$\begin{array}{ccc} N_{h} & = & S_{h}+E_{h}+A_{h}+I_{h1}+I_{h2}+R_{h}\;\text{and} \end{array}$$

$$\begin{array}{ccc} N_{v} & = & S_{v}+E_{v}+I_{v}, \end{array}$$

where *N*_*h*_ and *N*_*v*_ represent the total number of hosts and vectors respectively, of which *N*_*v*_ is time-dependent. The population of the Northeastern (NE) region of Brazil in 2014 was *N*_*h*_ = 56.7 million [52].

As in our previous work, it is assumed that the total mosquito population is given by:

$$\begin{array}{c} N_{v}\left(t\right)=m\left(t\right)\cdot N_{h}, \end{array}$$ (2)

where *m*(*t*) is the (time-dependent) ratio of mosquitoes population (*N*_*v*_(*t*)) to humans population (*N*_*h*_). In the model simulation, in order to reflect the changing dynamics of *m*(*t*), we increase the susceptible mosquitoes appropriately when *m*(*t*) increases, and remove the susceptible and infectious mosquitoes when *m*(*t*) decreases to compensate. In other words, the human population (*N*_*h*_) is fixed to be constant, whereas we vary the mosquito population (*N*_*v*_(*t*)) to reconstruct the time-dependent *m*(*t*).

#### Basic reproduction number

Following previous studies, the basic reproduction number, $R_{0}$, is derived using the next generation matrix method [6, 53, 54, 55]. We have

$$\begin{array}{c} R_{0}=\frac{R_{h}+\sqrt{R_{h}^{2}+4R_{v}^{2}}}{2}, \end{array}$$ (3)

where

$$\begin{array}{c} R_{h}=\beta \cdot [\eta \cdot \frac{1-\theta }{\gamma_{h}}+\theta \cdot (\frac{1}{\gamma_{h1}}+\frac{\tau }{\gamma_{h2}})], \end{array}$$

and

$$\begin{array}{c} R_{v}=a\cdot \sqrt{bcm\cdot \frac{\theta \gamma_{h}+\left(1-\theta \right)\eta \gamma_{h1}}{\gamma_{h}\gamma_{h1}}\cdot \frac{\sigma_{v}}{\mu_{v}\cdot \left(\mu_{v}+\sigma_{v}\right)}}. \end{array}$$

From Eq (3), it can be seen that $R_{0}$ depends on the mosquito-borne transmission path (in term of $R_{v}$) and the human-to-human transmission path (in term of $R_{h}$). Furthermore, if one excludes the exposed and asymptomatic compartments, ${\text{lim}}_{R_{h}\to 0^{+}}R_{0}=R_{v}=a\cdot \sqrt{\frac{bcm}{\gamma_{h1}\mu_{v}}}$, which provides the basic reproduction number of the classical Ross-Macdonald malaria model [6, 56, 57].

#### Model fitting and parameter estimation

To evaluate our methodology, model (1) was set up to fit the real epidemic data in NE Brazil. The time series of the number of weekly excess GBS cases in NE Brazil from Fig 2 (not Fig 1) is modelled as a partially observed Markov process (POMP, also know as hidden Markov model) with a “spillover” rate (*ρ*) from local symptomatic ZIKV cases. Here *ρ* is the combined effect of the GBS reporting ratio and the risk rate of symptomatic ZIKV inducing GBS i.e., the ratio $\rho =\frac{\text{reported}\;\text{GBS}}{\text{symptomatic}\;\text{ZIKV}}$ (see Table 1).

The simulated (weekly) number of excess GBS cases (*Z*_GBS_) from model (1) is considered as the theoretical or true number of cases. And the corresponding observed GBS cases of the *i*-th week, $C_{\text{GBS}}^{\left(i\right)}$, are assumed to have a Negative-Binomial (NB) distribution [6, 41, 44, 58, 59, 60].

$$\begin{array}{c} C_{\text{GBS}}^{\left(i\right)}∼\text{NB}(n=\frac{1}{\tau },\;p=\frac{1}{1+\tau Z_{\text{GBS}}^{\left(i\right)}})\;\text{with}\;\text{mean:}\;\mu_{i}=Z_{\text{GBS}}^{\left(i\right)}. \end{array}$$ (4)

Here, *τ* denotes an over-dispersion parameter that needs to be estimated. Finally, the overall log-likelihood function, *ℓ*, is given by

$$\begin{array}{c} \ell (\Theta |C_{\text{GBS}}^{\left(1\right)},\ldots ,C_{\text{GBS}}^{\left(N\right)})=\sum_{i=1}^{T}\text{log}\;[L_{i}(C_{\text{GBS}}^{\left(N\right)}|C_{\text{GBS}}^{\left(1\right)},\ldots ,C_{\text{GBS}}^{\left(i-1\right)};\Theta )]. \end{array}$$ (5)

The vector Θ denotes the parameter vector under estimation. The *L*_*i*_(⋅) is the likelihood function associated with the *i*-th NB prior defined in Eq (4). The term *T* denotes the total number of weeks during the study period.

Our methodology reconstructs the mosquito abundance *m* = *m*(*t*) which is otherwise unknown but variable and time-dependent over the study period. Following Eq (3), the basic reproduction number is a function of *m*(*t*), and thus we also allow $R_{0}$ to be time-dependent (i.e., $R_{0}=R_{0}\left(t\right)$). The time-dependent *m*(*t*) is climate-driven and modelled as an exponential function of the daily average temperature and rainfall time series, together with a two-piece step function for the baseline component. It is modelled as follows

$$\begin{array}{c} \begin{array}{cc} m(t) & =m(t;\tau_{0},\tau_{1},p_{1},p_{2},p_{3},p_{4})\\ & =\text{exp}[p_{1}\text{Temperature}\left(t-\tau_{0}\right)+p_{2}\text{Rainfall}\left(t-\tau_{0}\right)+p_{3}1\left(t<\tau_{1}\right)+p_{4}1\left(t⩾\tau_{1}\right)]. \end{array} \end{array}$$ (6)

The term *τ*_0_ is the time delay between the occurrence of weather factors and their effects on the GBS epidemic. It contains the lagged effect on the local mosquito population, the progress from ZIKV to GBS development and any reporting delay. Previous studies [26, 61] suggest there exists a time delay of at least 3 weeks between the exposure of patients to ZIKV and the development of GBS (i.e., an incubation period plus a typical reporting delay). For the mosquito, the life cycle progresses from an egg to an adult, and maturity takes approximately 8-10 days [62]. Therefore, the time lag of the effects from the weather factors are taken to be one month in total i.e., *τ*_0_ = 3 × 7 + (8 + 10)/2 = 30 days.

In Eq (6), *p*_1_ and *p*_2_ are the scale parameters controlling the effects of local temperature and rainfall respectively. The two terms *p*_3_ and *p*_4_, are time-driven baseline effects characterizing trends in *m* that switch on depending on the time period *τ*_1_. The parameter *τ*_1_ could be viewed as the timing of baseline change in the mosquito population, due to the possible interference between ZIKV and CHIKV for instance, and/or local mosquito control measures. The function **1**(⋅) is an indicator function, which equals 1, if the condition in the brackets is true; but is 0 otherwise.

Based on fitting and comparisons, the scale of *p*_2_ was found to be negligible in magnitude, indicating that the effects of the local rainfall is negligible compared to temperature. Thus, in most parts of the analysis that follows, we neglect the rainfall term in Eq (6) for simplicity.

Bellan (2010) [49] argued that the mosquito lifespan is 30 days on average but under control and/or age dependent mortality the lifespan is much shorter. For this reason Kraemer et al (2017) [63] implemented a mosquito lifespan of average 7 days but with a maximum of 30 days. However, according to the results of Bellan (2010) [49] this would correspond to a high level of mosquito control or age-dependence. We believe a more appropriate (less extreme) choice for the NE Brazil context would correspond to an average 14 day mosquito lifespan.

According to Eq (3), $R_{0}$ is a function of *m*(*t*) and thus $R_{0}$ is also time-dependent. Hence, $R_{0}$ can also be determined by the parameters in Eq (6), i.e., $R_{0}=R_{0}\left(m\right)=R_{0}\left(t;\tau_{0},\tau_{1},p_{1},p_{2},p_{3},p_{4}\right)$. Besides the climate-driven model, we also test a non-mechanistic model where the mosquito population (or transmission rate) is an exponential of a cubic spline function (S1 Text). Similar techniques were used in our previous work [44]. We compare the result with the climate-driven model and the non-mechanistic model.

The parameter fitting and inference process are rigorously and exhaustively checked within biologically and clinically reasonable ranges. We should have confidence that the fits of observed time-series are realistic because of the consistency with the true underlying epidemiological processes rather than because of artificial model over-fitting. The maximum likelihood estimate (MLE) approach is adopted for model parameter estimation. The 95% confidence intervals (CI) of parameters are estimated based on the parameter ranges in Table 1, using the method of profile likelihood confidence intervals [40, 41]. More details for finding the uncertainty of $R_{0}\left(t\right)$ estimation is in S1 Text.

The Bayesian Information Criterion (BIC) is employed as a criterion for model comparison, and quantifies the trade-off between the goodness-of-fit of a model and its complexity [64]. The simulations were conducted by deploying the Euler-multinomial integration method with the time-step fixed to one day [41, 56]. We deploy iterated filtering and plug-and-play likelihood-based inference frameworks to fit the reported number of excess GBS cases time series [6, 41, 44, 65, 66]. Since a plug-and-play inference framework has been adopted, our model (simulation scheme) includes both demographic noise (Euler-multinomial type) and measurement noise (negative binomial). The deterministic model we analyse in the main results is the mean field model of the stochastic version. The inference scheme which infers model parameters, requires that the underlying model is stochastic, which is the case here. The R package “POMP” is available via [67]. Parameter estimation and statistical analysis are conducted by using R (version 3.3.3) [68], and the equations are written in C programing language (see S1 Text).

## Results

### Connecting the GBS and ZIKV data, and changing reporting rates

Fig 1 plots the time series of ZIKV cases and GBS from the period April 1 of 2015 to March 31 of 2016. The data are an aggregation of the six countries Columbia, the Dominican Republic, El Salvador, Honduras, Suriname, and Venezuela as well as the Bahia State in Brazil. These time series demonstrate the tight connection between the reported ZIKV disease and GBS, whose case numbers closely mimic one another in time. The connection is the basis of our method for estimating ZIKV cases from GBS reports, which as we have discussed, are by their nature, reasonably reliable records.

The Northeastern Brazil datasets which are analysed in this paper are plotted in Fig 2. The time series indicate two epidemic outbreaks of reported ZIKV cases, where the second outbreak in 2016 is far stronger than the first in 2015. Despite this, the two waves of GBS appear similar over the two years, although a close examination reveals there were fewer cases in 2016. If one ignores the possible regional difference and adopts the GBS-ZIKV risk rate of 0.032% i.e., 0.32 GBS cases per 1,000 ZIKV infections (asymptomatic and symptomatic) calculated in [23], the total cases of ZIKV can be approximated according to the excess GBS cases time series (Fig 2). But this is a naive calculation and we will seek ways to improve this.

Tallying the case numbers, in 2015 there were 233 excess GBS cases and 38,641 reported ZIKV cases, but in 2016 there were 168 excess GBS cases and 70,916 reported ZIKV cases. The ratio of GBS/Zika reported cases is plotted (blue) in Fig 2, and one sees the transition from GBS/ZIKV(repoted) (= 233÷38641 = 0.60% in the first year (2015) to GBS/ZIKV(reported) =168÷70916 = 0.24% in the second year (2016).

Let us first assume that the GBS/ZIKV (reported) ratio did not change in time in any major way over the two years 2015 and 2016. Our analysis of data from the time series in Fig 2 shows that as GBS cases dropped from 233 cases in 2015, to 168 cases in 2016, i.e. by a factor of 0.72 (168/233), the number of reported ZIKV cases rose by a factor of 70, 916÷38, 641 = 1.8. The only explanation for this is that there must have been a major under-reporting of ZIKV cases in the first year of 2015 [60, 69]. This also seems reasonable since in 2015 the official WHO ZIKV reporting program had not yet been launched [20]. Suppose now the GBS/ZIKV(reported) ratio was 0.24% in both 2015 and 2016 even though we know that this could not be the case. A simple calculations shows that there should have been some 98,353 (= 233 × 70916÷168) ZIKV reported cases in 2015 rather than only the 38,641 cases that were reported in reality. Thus for the 2015 year, it would appear that ZIKV was under-reported by a factor of 2.5 when compared to the ZIKV reporting rate in 2016.

### Fitting the model to GBS data

We fit model (1) based on the reported excess GBS cases time series shown by the dark blue dotted line in Fig 2. This was repeated for different sets of baseline parameters, with several different (possible) values of *η* (asymptomatic ZIKV relative infectivity) and *θ* (proportion of symptomatic ZIKV infections) considered. The *θ* = 0.5 simulations correspond to a 1:1 ratio of the symptomatic to asymptomatic ZIKV infection of [22]. Simulations with *θ* = 0.2 correspond to the 4:1 ratio of the symptomatic to asymptomatic ZIKV infection of [11].

**Fig 4:**
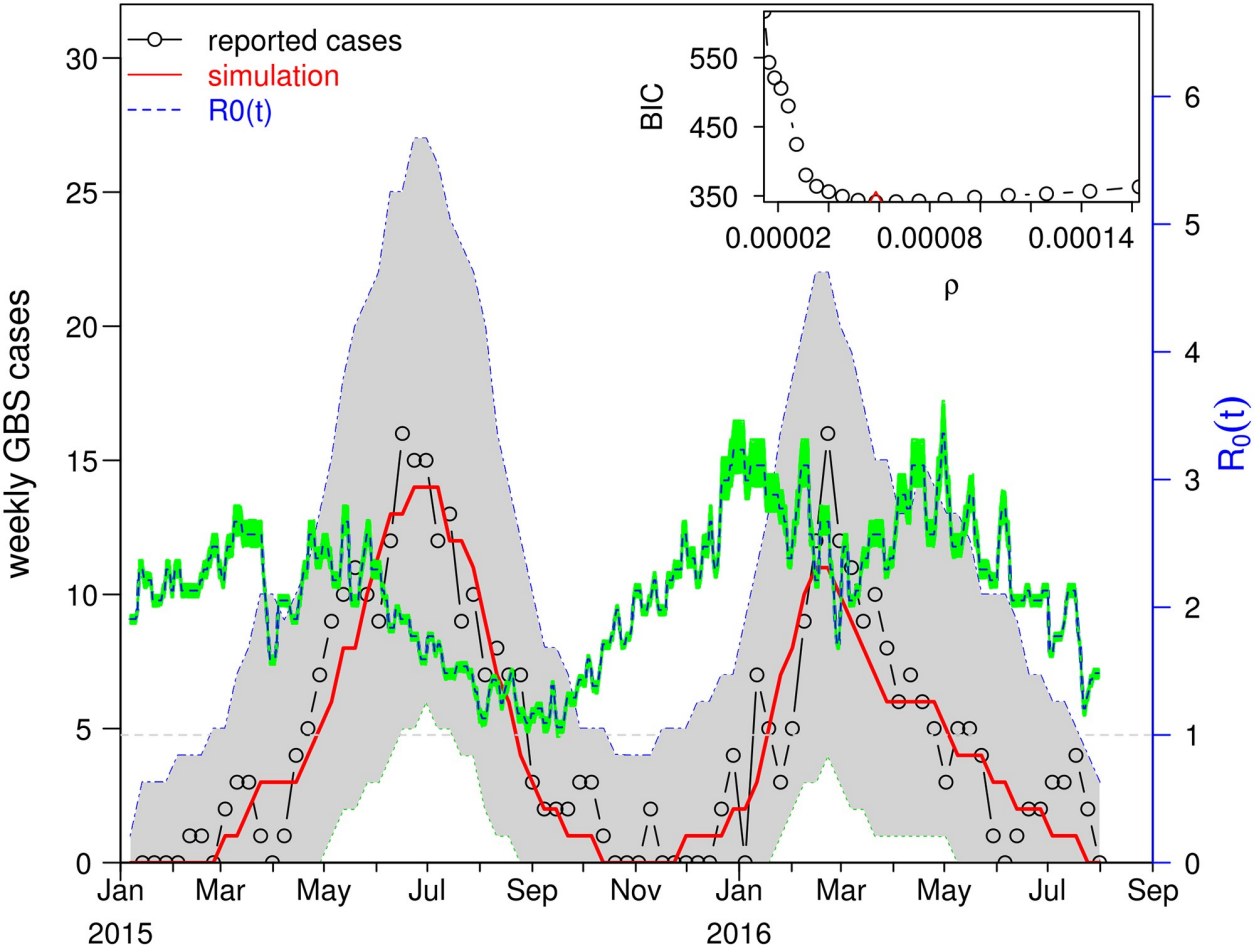
The fitting results for *θ* = 0.5 and *η* = 0.3. The fitting results in the main panel show the best scenario, which attains the smallest BIC. The red line is the mean GBS cases averaged from 1000 simulations plotted as a function of time. The dashed blue curve is the $R_{0}\left(t\right)$ estimate which minimizes the BIC, and the green region shows the 95% of confidence interval $R_{0}\left(t\right)$. The grey shaded area shows the 95% CI of the simulated GBS cases. The inset panel shows the profile of BIC as a function of *ρ*. The minimum occurs at *ρ* = 0.000061, which is our best estimate for *ρ*.

Fig 4 shows the fitting results with *θ* = 0.5 and *η* = 0.3. The mean GBS values for 1000 simulations are plotted in time (red) and fit the trajectory of the reported GBS cases (black line) closely. The grey shading gives the 95% credible interval (CI) of the case numbers for each day of the simulation. The model fits the data well, and all 95% CI cover the associated observations. This indicates the simulation outcomes are not statistically different from the observations, and thus our model successfully reconstructed the two waves of the ZIKV epidemic in NE Brazil. Since we adopted a Negative Binomial type measurement noise in Eq (4), the weekly simulated cases have a variance larger than the mean. Thus, the simulation has a wide CI. We estimate the time-dependent $R_{0}\left(t\right)$ which ranged from 1.1 to 3.3 over the whole study period. In Fig 4, the green region denotes the 95% CI of $R_{0}\left(t\right)$. For the detailed method, please see S1 Text. The simulations determine the best fitting initial condition of susceptible population is *S*_*h*_(0) = 0.55. The inserted panel shows the parameter estimation of *ρ* found where the likelihood profile reaches the minimum BIC value. Namely, we fix *ρ* at 20 values over a range, fit the model (1) to the GBS data, and calculate the BIC. While the minimum is *ρ* = 0.00061, a value of *ρ* from 0.00005 to 0.0001 will yield an (almost) equivalent level of BIC given the flatness of the curve in this regime.

In addition to the mechanistic reconstruction of $R_{0}\left(t\right)$ in the main results here, we also present a non-mechanistic reconstruction in S1 Text. The non-mechanistic approach is implemented by using a cubic spline function to reconstruct $R_{0}\left(t\right)$. The model also fits the disease surveillance data well. The BIC of the non-mechanistic model is 7 units larger than the above climate-driven model in Fig 4. We find that the non-mechanistic reconstruction of $R_{0}$ matches the daily temperature reasonably well. This suggests the weather-driven $R_{0}\left(t\right)$ in our main results here is neither coincidental nor artificial.

### Estimation of the infection attack rate (IAR) and model parameters

The estimates of the GBS/ZIKV ratio *ρ* and the IAR are summarised in Table 2. For the ZIKV symptomatic ratio parameter, *θ*, we follow the previous serological study conducted in French Polynesia that found the asymptomatic: symptomatic case ratio as 1: 1 in the general population [22]. Thus, *θ* is set at *θ* = 0.5 for the scenarios in the main results section. But with thus setting (*θ* = 0.5), the estimation of *ρ* is found to sit roughly at *ρ* = 0.000063 (= 0.0063%). This appears to hold even if *η*, the relative infectivity of the asymptomatics, is changed over the interval (0, 1). Estimates of *ρ* thus appear to be reasonably insensitive to the change of relative infectivity of the asymptomatics (*η*). However, *ρ* is sensitive to the change of the symptomatic proportion of ZIKV infections (*θ*). Setting *θ* = 0.2 gives *ρ* = 0.00013, but as Table 2 reveals, this result is also relatively insensitive to changes in *η*.

**Table 2:** Summary table of the estimation results of *ρ* and IAR. The estimates with *θ* = 0.5 and *η* = 0.3 are used as main results, also in Fig 4.

*θ*	*η*	*ρ*	95% CI	IAR	95% CI
0.5	0.1	0.000053	(0.000046,0.000080)	0.2792	(0.1841, 0.3234)
0.5	0.3	0.000061	(0.000050,0.000086)	0.2411	(0.1711, 0.2932)
0.5	0.5	0.000063	(0.000049,0.000086)	0.2352	(0.1711, 0.3005)
0.5	0.7	0.000063	(0.000050,0.000084)	0.2352	(0.1753, 0.2932)
0.5	0.9	0.000067	(0.000053,0.000086)	0.2186	(0.1711, 0.2792)
0.2	0.1	0.000139	(0.000083,0.000169)	0.2645	(0.2175, 0.4423)
0.2	0.3	0.000129	(0.000117,0.000178)	0.2847	(0.2071, 0.3140)

To calculate the number of ZIKV cases and the IAR, we use the estimate obtained for *ρ* (*ρ* = 0.00061—ratio of reported GBS to symptomatic ZIKV), and denote the ZIKV symptomatic ratio as *θ*, as before. *ρ* can be estimated from the model. Then, the number of ZIKV cases equals (the number of reported GBS) ÷ [reported GBS/symptomatic ZIKV] ÷ (ZIKV symptomatic ratio), which is the number of the reported GBS/*ρ*/*θ*. The IAR equals the number ZIKV cases ÷ the total population in the NE Brazil.

For all pairs of *θ* and *η* in Table 2, the estimated IARs are similar with IAR varying approximately from 22% to 28% and the 95% CIs largely overlap. Thus, for *θ* = 0.5, we can be at least 95% sure the IAR of the ZIKV epidemic is below 33%, and is likely to be well below.

The estimates of the initial susceptible levels (*S*_*h*_(0)) and the parameters (*p*_1_, *p*_3_ and *p*_4_) that control the temporal pattern of $R_{0}\left(t\right)$ are summarised in Table 3. Note that according to Eq (3), *m* is proportional to $R_{v}^{2}$ (i.e., $m\propto R_{v}^{2}$), a key term in the formula for the basic reproduction number. It is not hard to show that [exp(0.5*p*_1_) − 1] × 100% is the change rate in $R_{v}$ when there is one unit (°C) increase in temperature. From Table 3, one unit increase in temperature will lead to an increase of (exp(0.5 × 0.52) − 1 =) 29.7% in $R_{v}$ when *η* = 0.1. And one unit increase in temperature will lead to (exp(0.5 × 0.53) − 1 =) 30.3% increase in $R_{v}$ when *η* = 0.3. Eq (3) shows the $R_{0}$ is comprised of $R_{v}$ and $R_{h}$, where the $R_{h}$ is the contribution from the sexual transmission path. The sexual transmissibility of ZIKV can be ignored owing to (i) the contribution of this path is negligibly small [6, 7]; and (ii) the recommendation to abstain from sexual contact during ZIKV epidemics [10]. Hence, the term $R_{h}$ could be very close to zero, and its contribution to the whole $R_{0}$ is probably far less than the mosquito-borne transmission $R_{v}$. According to Eq (3), when $R_{h}=0$, then $R_{0}=R_{v}$. Provided ${\text{lim}}_{R_{h}\to 0^{+}}R_{0}=R_{v}$, the effect of the temperature on $R_{v}$, is determined by the estimate for *p*_1_ estimate, is (almost) equivalently applicable to $R_{0}$.

**Table 3:** Summary table of the estimation results of the initial susceptibility (*S*_*h*_(0)) and parameters *p*_1_, *p*_3_ and *p*_4_ in Eq (6). The estimates with *θ* = 0.5 and *η* = 0.3 are used as main results, also in Fig 4.

*θ*	*η*	*S*_*h*_(0)	95% CI	*p*_1_	95% CI	*p*_3_	95% CI	*p*_4_	95% CI
0.5	0.1	0.55	(0.47,0.73)	0.52	(0.44,0.63)	0.53	(0.40,0.67)	0.25	(0.16,0.37)
0.5	0.3	0.57	(0.46,0.74)	0.53	(0.44,0.63)	0.44	(0.34,0.55)	0.21	(0.13,0.31)

In Table 3, the initial susceptibility *S*_*h*_(0) is estimated to be 0.55 (95% CI: 0.47-0.73) when *η* = 0.1, and 0.57 (95% CI: 0.46-0.74) when *η* = 0.3. The large overlap in the 95% CIs indicates that the two *S*_*h*_(0) estimates are not statistically different. According to the 95% CIs of *S*_*h*_(0), it is likely that over a quarter (i.e., > 25%) of the whole population were not involved in the 2015-16 ZIKV epidemic.

The time points (*τ*_1_) when the baseline of *m*(*t*) (or $R_{0}\left(t\right)$) changes from *p*_3_ to *p*_4_ in Eq (6) may also be estimated. It was found that *τ*_1_ is most likely to be March 7 of 2016. For the parameters *p*_3_ and *p*_4_, we find significant difference in the baseline levels of *m*, which suggested the existence of the non-weather-driven temporal changes in the ZIKV transmissibility.

### Results of the sensitivity analysis

As is conventional, the Partial Rank Correlation Coefficients (PRCC) are adopted to perform a sensitivity analysis of the model [6, 44, 66, 70, 71]. Firstly, 1000 random samples are taken from uniform distributions of each model parameter. The ranges as set out in Table 1. Secondly, for every random parameter sample set, the ZIKV-GBS model was simulated to obtain the target biological quantities (e.g., $R_{0}$ and total number of GBS cases in this study). Finally, PRCCs were calculated between each parameter and target biological quantities.

**Fig 5:**
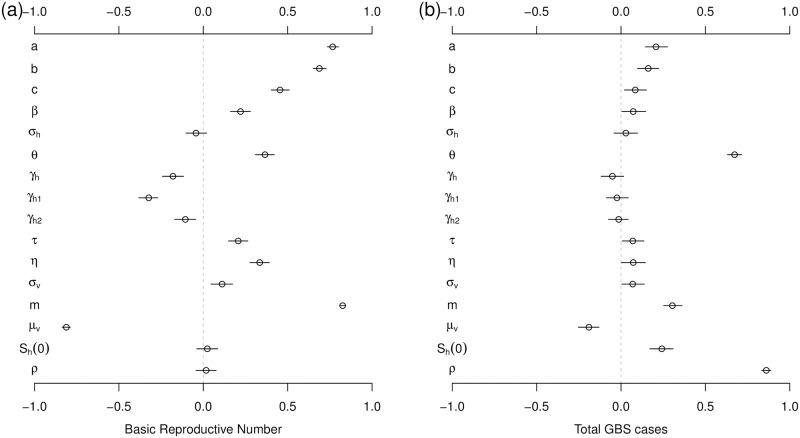
The Partial Rank Correlation Coefficients (PRCC) of the basic reproduction number, $R_{0}$, (panel (a)) and total GBS cases (panel (b)) with respect to model parameters. The *S*_*h*_(0) in this figure denotes the initial susceptible ratio, i.e, *S*_*h*_(0)/*N*_*h*_. The black circle is the estimated correlation, and the bar represents 95% CI. The ranges of parameters are in Table 1.

Results of the sensitivity analysis are presented in Fig 5, which indicates how model parameters impact the basic reproduction number ($R_{0}$) and the total reported GBS cases. $R_{0}$ is most sensitive to the vector’s biting rate (*a*), the vector to host ratio (*m*) and the vectors’ lifespan ($\mu_{v}^{-1}$, or vectors’ natural death rate, *μ*_*v*_), indicating the importance of the mosquitoes role in disease transmission. The total reported GBS cases are considerably sensitive to the proportion of symptomatic cases (*θ*), and the ratio (or risk) of excess GBS cases to symptomatic ZIKV infections (*ρ*).

## Discussion

The epidemiological modelling approach used in this paper (Eq (1)), is based on the assumption that populations are mixing homogeneously and climate variables are spatially homogeneous over NE Brazil. We discuss our justification in using these assumptions in great depth in S1 Text. The assumption of mixing populations in a region this size is often considered a workable approximation for epidemiological models given modern commuting patterns, given the robustness of the SIR modelling approach, and given the ability of an infection to rapidly spread through a large set of cities that are even weakly connected within a country. The same approach has been used in many related papers and appears to be successful when used with care. Previous studies in large populations, such as measles in England and Wales (population size 50 million [72, 73]) cholera in Yemen (population size 27 million [74]), have yielded acceptable results. Our modelling framework (the temperature function and using GBS as a proxy for ZIKV) would work better if more data were available at the city or state level, but currently this is not obtainable.

A careful discussion on the limitations of aggregating the weather data, as used in Eq (6) is given in S1 Text. There we explain that our approach in which data is spatially aggregated was inspired from the success of de Oliveira *et al*. (2017) [26] in studying Zika virus and its impact on human populations including microcephaly numbers and its relation to GBS. Their analysis heavily relies on the property that there is coherence in the dynamics of infectives and microcephaly populations when aggregated over all NE Brazil. That is the spatially averaged data generates a coherent and meaningful signal in time. We also demonstrate that spatial climate homogeneity, the assumption behind spatial aggregation, is a reasonable approximation according to recent literature on the climate distribution of NE Brazil.

Nevertheless, we explain that the city of Petrolina is an outlier in terms of its high Summer temperatures (which could have major impact on the mosquito dynamics), and this is why spatial aggregation needs to be performed with care and with caution. Checks need to be made with historical data to show that the climate variables are at least approximately spatially uniform, as was found to be the case here. Finally, we point out that aggregation of data is a commonly used approach (when it can be justified) for epidemiological analyses at the country level for many regions in the world ([72, 12], including NE Brazil ([12, 26].

Based on the parallel between cases of ZIKV disease and cases of GBS (eg., as seen in Fig 1), we have proposed a ZIKV model that is calibrated on case data of GBS. ZIKV case numbers are obtained by scaling up from GBS. The advantage of this practice is that the GBS case numbers are more trustworthy and reliable compared to numbers obtained through surveillance of ZIKV where there is much scope for errors in the reporting rate. Our model considers heterogeneity in symptomatic and asymptomatic ZIKV infections (i.e., *θ* and *η*) as well as the local mosquito population (*m*). Model (1) was fitted to the reported excess GBS cases time series with different sets of parameters for symptomatic proportion (*θ*) and asymptomatic infectivity (*η*).

From a recent metadata study [23] and a serological study [22], the ratio *ρ* of GBS to symptomatic ZIKV cases was found to be 0.00024 and 0.00032 respectively (see Introduction of this study). Similarly, based on the data from eleven countries, Mier-y-Teran-Romero *et al*. [15] found the overall estimate for the risk of reported GBS “was 2.0 (95% CI 0.5-4.5) GBS reported cases per 10,000 ZIKV infections, i.e., 0.02%, (which is) close to the point estimate of 2.4 GBS cases per 10,000 ZIKV, i.e., 0.024%, infections estimated using only data from French Polynesia.” In this study, the model estimation finds a ratio between GBS and symptomatic ZIKV cases as *ρ* = 0.000061 or equivalently *ρ* = 0.0061% with 95% CI 0.0050%-0.0086%. That is, 1 GBS case per 16,400 ZIKV symptomatic cases which is approximately one quarter or 25% the magnitude of existing estimates. Our estimate, although still tentative and based on reasonable first approximations, seems plausible since ZIKV surveillance was generally unreliable and probably severely under-reported, especially before 2016 [60, 69]. For this reason, we avoided using the ZIKV surveillance data to fit the epidemic model, and our estimate of *ρ* depends on the more reliable GBS data.

We considered different values of *θ* (proportion of symptomatic cases) and *η* (infectivity scale of asymptomatic) in this study. It is worth noting that *η* is a parameter that was overlooked previously. The values of *θ* we used, and our estimated IAR are in line with previous studies, e.g. [75] in Martinique. According to Cousien *et al*. [75] as well as the key literature they refer to, “the proportion of asymptomatic infections (40% with 95%CI of 23-56%) was low compared with previous estimates from a Yap island outbreak (80%) or among pregnant women in French Guiana (77%). However, it was consistent with estimates obtained in a household investigation in Puerto Rico (43%), a sero-survey in French Polynesia (50%), a survey among blood donors Martinique (45%), and the reanalysis of surveillance data from French overseas territories (<50%).” Our current setting of *θ* = 0.5 and *θ* = 0.2, i.e., asymptomatic of 50% and 80% respectively, are consistent with these previous studies, and cover the indicated range.

The model analysis estimated the IAR of ZIKV cases in NE Brazil to lie between 22% to 28% for the two waves. This is based on the assumption that the proportion of symptomatics *θ* = 0.5, which appears to be reliable according to the serological results of Aubry *et al*. [22]. It is also in line with a number of models and empirical estimates for other areas of Brazil and South America. For example, Zhang *et al*. (2017) estimated some 18% IAR for the areas in Brazil [12]. In pointing this out, we must also note that most IAR estimates in the literature need to be treated with caution. Due to poor surveillance, limited knowledge about the ZIKV reporting ratio and population seropositivity, the estimates may have been based on samples that were not representative of the general population as a whole. Previous estimates of IAR relied on poor ZIKV data in Brazilian regions: some estimates appear to be less than 20%, and others yield more than 50% (see S1 Text). However, as mentioned, all these estimates must be treated with caution. This study is the first to use the more reliable GBS data as a proxy to estimate the IAR of ZIKV epidemics. We found that the IAR is likely to be below 33% in the whole NE Brazil.

de Oliveira *et al*. [26] also identified a striking relationship between the dynamics in time of the first wave of excess GBS and that of microcephaly. Their Fig 1B shows the dynamics in a time of these two conditions are almost identical apart from a delay of 23 weeks and differing otherwise by a scale factor. The remarkable similarity in the different epidemic time series allows us to compare the rates of GBS cases to those of microcephaly. By examining the peak heights of the two diseases, the ratio between them is 6.1 (maximum of microcephaly divided by maximum of GBS wave), which corresponds to 1 GBS case for every 6.1 microcephaly cases. If we make the reasonable assumption that the reporting rate of both conditions is similar, it is clear that GBS is a much rarer disease than microcephaly. Nevertheless, we still chose to predict ZIKV cases based on GBS rather than microcephaly cases, because of problems in the correct reporting of microcephaly over the study period. For example, the criteria for identifying microcephaly changed dramatically at different times over the two year period and in different areas, making the reporting coverage highly unstable. Moreover, previous to this period, reporting was not compulsory nor was there consistently defined criteria for identifying the condition.

Return now to the dynamics of the reconstructed ZIKV cases generated by Eq (1) as calibrated on the GBS data (Fig 4). The reproductive number, $R_{0}\left(t\right)$, which quantifies the transmission rate, was reconstructed by modelling the local meteorological data with Eq (6). The estimated $R_{0}\left(t\right)$ was found to oscillate due to seasonality between the values $1.1<R_{0}<3.3$, and on average was found $\langle R_{0}\rangle =2.2$. The average level and estimated range of $R_{0}\left(t\right)$ are in line with previous studies [12, 60, 69]. Because of temperature dependence, $R_{0}\left(t\right)$ reached minimum values in winters. The range of values the model predicted for $R_{0}\left(t\right)$ is very similar to the intensities reported in Fig 3 of [12] for ZIKV in Brazil.

As the net growth rate of mosquitoes tends to increase as temperature increases [12, 76, 77], it is not surprising that our estimated *p*_1_ > 0 (the temperature dependence parameter in *m*(*t*)) is positive. The positive association between temperature and transmissibility has also been observed in the literature [69]. Significant nonzero estimates were found for parameters *p*_3_ and *p*_4_, which also control *m*(*t*), and thus the reproductive number $R_{0}$. This immediately suggests the existence of non-weather-driven temporal changes in the ZIKV transmissibility. The baseline drop in *m*(*t*) would also lead to a drop in $R_{0}\left(t\right)$, and indicates a decrease in ZIKV transmissibility across the two epidemic waves. Since the official mandatory ZIKV reporting began in February 2016, this may have increased public awareness of ZIKV risk, and thus reduced the spread of infection [70, 78, 79, 80, 81]. Disease control measures were also introduced by some local authorities during the second epidemic wave. The time-change point (*τ*_1_) when the baseline *p*_3_ switches to *p*_4_ in the model corresponds to March 7 of 2016. Interestingly, this time point coincides with the peak timing of the concurrent CHIKV outbreak [26]. Also, very close to this date, $R_{0}\left(t\right)$ passed through a local minimum and then increased for a two month period, generating, in turn, an increase in GBS cases.

We compared the results of a non-mechanistic model in S1 Text which did not take into account climatic factors, and those from the climate-driven model in Fig 4. Although the non-mechanistic model did not perform as well, it nevertheless provided useful insights by producing results that matched the impact of the daily temperature on $R_{0}$, the transmission of ZIKV.

Continuing further, we now attempt to estimate the reporting rate of ZIKV. We argue that the reporting rate of ZIKV disease increased dramatically around February and March of 2016, as suggested also in the literature [69]. Thus, it is reasonable to assume that the data for the second wave of ZIKV in 2016 is more reliable than that of the first. Taking the maximum of the second ZIKV wave divided by the maximum of the GBS wave, we find the ratio between the two diseases is 436; i.e., 1 GBS case per 436 reported ZIKV cases. However, our model fitting finds a ratio between GBS and symptomatic ZIKV cases as *ρ* = 0.000061, or 1 GBS case per 16,400 ZIKV symptomatic cases. Thus, we can conclude that the reporting ratio of symptomatic ZIKV cases is roughly 16, 400/436 ≈ 38. Namely, for every 38 symptomatic ZIKV cases, there was 1 case reported, over the second wave in 2016. Hence we arrive at an estimate for the reporting ratio of ZIKV, namely 1:38. Moreover, as mentioned, when taking this reporting ratio into account our estimated IAR falls in the reasonable range 22% to 28% for the two waves.

Results in Table 3 indicate that the initial population susceptibility, *S*_*h*_(0), was likely below 0.75. There are several plausible explanations. First, it has recently come to light that a large proportion of the population was already exposed to dengue before the 2015 Zika outbreak and this may have provided a significant proportion of the population with cross-immunity [82]. As found in Rodriguez-Barraquer *et al*. [82] the “pre-existing high antibody titers to dengue virus were associated with reduced risk of ZIKV infection and symptoms.” A similar outcome was noted in Gordon *et al*. [83]. Second, Faria *et al*. [84] indicated that the Zika virus (Asian ZIKV genotype) may already have been in Brazil as early as 2013, and thus, there may have been some exposure and build up of immunity by 2016. However, given the almost nonexistent reports of ZIKV, it is unlikely that this generated any major population immunity. Third, there exists significant spatial heterogeneity in the Brazilian population distribution [35]. Messina *et al*. [85] reported that most of the ZIKV incidences of NE Brazil were distributed in the coastal areas, and thus population in the inland areas were likely spared from the epidemic. This may also contribute to our estimated initial susceptibility.

## Supporting information

S1 Text Supplementary information. (PDF)
