# Supplementary material for: New estimates of the Zika virus epidemic attack rate in Northeastern Brazil from 2015 to 2016: A modelling analysis based on Guillain-Barré Syndrome (GBS) surveillance data
Source: PLoS Negl Trop Dis. 2020 Apr 29;14(4):e0007502. doi: 10.1371/journal.pntd.0007502 (PMC7213748; doi:10.1371/journal.pntd.0007502)
Supplement: S1 Text — (PDF) [file pntd.0007502.s001.pdf]

Supplementary Information

Daihai He[1,*], Shi Zhao[1,2,3], Qianying Lin[1,4], Salihu S. Musa[1] & Lewi Stone[5,6,*]

1 Department of Applied Mathematics, Hong Kong Polytechnic University, Hong Kong, China

2 Division of Biostatistics, JC School of Public Health and Primary Care, Chinese University of Hong Kong, Hong Kong, China

3 Clinical Trials and Biostatistics Lab, Shenzhen Research Institute, Chinese University of Hong Kong, Shenzhen, China

4 Michigan Institute for Data Science, University of Michigan, Ann Arbor, Michigan, US

5 Mathematical Science, School of Science, RMIT University, Melbourne, Vic., Australia

6 Biomathematics Unit, School of Zoology, Faculty of Life Sciences, Tel Aviv University, Tel Aviv, Israel

∗ Correspondence to: daihai.he@polyu.edu.hk (D.H.), and lewistone100@gmail.com (L.S.)

April 25, 2020

Contents

S1 Text **2**
S1.1 Case Definition of the Guillain-Barré Syndrome 2
S1.2 Justification for data aggregation 3
S1.3 Uncertainty in reproduction number 8
S1.4 Fitting Results with Cubic Spline Reconstruction (i.e., no climate variables involved) . 9
S1.5 Brief Review of the Zika epidemics Infection Attack Rate 11
S1.6 Code of the model equations and simulation scheme 11

S1 Text

S1.1 Case Definition of the Guillain-Barré Syndrome

The Guillain-Barré Syndrome (GBS) time series of cases in NE Brazil were kindly provided by Professor Oliveira, as used in the recent study in [1]. Hence, the GBS cases are confirmed following the case definition protocol [1, 2].

Suspected case was defined by "*patient with neurological manifestation of undetermined origin who attended a sentinel unit (SU), and with a previous viral infection up to 60 days before the onset of neurological symptoms. Neurological manifestations include: encephalitis, meningoencephalitis, myelitis, acute flaccid paralysis, acute disseminated encephalomyelitis (ADEM) and/or Guillain-Barre syndrome. Here the SUs are hospitals only, and not necessarily the same SUs as used for Zika case detection.*"

Probable case was defined by "*suspected case with clinical signs of Zika virus infection, dengue or chikungunya, without laboratory confirmation.*"

Confirmed case was defined by "*suspected case with laboratory confirmation by RT-PCR for the following etiological agents: Zika (samples of cerebrospinal fluid, i.e., CSF, urine or serum), Dengue (CSF or serum samples), Chikungunya (CSF or serum samples).*"

The excess GBS used in our work were calculated by "*subtracting from the averages for corresponding weeks in each of the 5 years from 2010-14.*"

S1.2 Justification for data aggregation

Brazil has a territory of more than 8.5 million km^2 and is one of the largest countries in the world with an estimated population of 211 million. The North East region of Brazil is one of five regions, and has an area of 1.6 million km^2. The region has a population of 57 million, but 75% of the population lives in the major cities of the coastal area which stops at the Atlantic on the East. While larger Brazil does have spatially heterogeneous climate trends, a large part of the heterogeneity can be attributed to its five relatively homogeneous climate regions. As Hansel et al. (2016) write: "The Brazilian territory has a wide climate diversity ... and can be classified into five major areas:

(1) the south (S) is dominated by a temperate climate of low diversity, but experiences notable thermal fluctuations throughout the year;

(2) the southeast (SE) is a transitional region between the warm climates of low latitudes and the temperate climate of middle latitudes;

(3) the central west (CW) presents a domain of high temperatures in spring and summer and mild in winter;

(4) the northeast (NE) is where high temperatures are predominant throughout the year, but with two major climatic types (hot and warm); and

(5) the northern region (N) is always hot, without seasonal temperature fluctuation."

This is a well-known division. See also: Alvares et al 2013 "Köppen's climate classification map for Brazil" for a discussion of climate divisions.

We are interested specifically in North East Brazil which is the fourth of these climate zones. Temporally NE Brazil has a known seasonal cycle in terms of climate variables (temperature, rainfall) that impresses itself over all the region, and is relatively spatially uniform especially over the coastal areas where 75% of the population reside. Spatially speaking, the relative climate homogeneity is evident from studying the data for six coastal cities given in Hänsel et al (2016) as displayed in the graphs given in Fig A (two left-hand columns). The seasonal temperature trends (black lines with grey standard deviations) are extremely similar in all these different cities, as can be seen by comparing the trends down any column. The annual seasonal rainfall trends are also similar across these cities according to the same graphs (second column from left). However, rainfall is expected to be more heterogeneous, given erratic El Nino rain events have significant influence.

Reasonable spatial uniformity of the seasonal temperature cycle in NE Brazilian cities is also confirmed from the spatial maps calculated by Alvares et al. (2013), which are reproduced below. For example, the left map of Fig B plots the maximum temperature experienced over all NE Brazil, and most of the region is of the same red colour indicating a large degree of spatial uniformity for this index.

[Reference] Hänsel S, Medeiros DM, Matschullat J, Petta RA, de Mendonça Silva I. Assessing homogeneity and climate variability of temperature and precipitation series in the capitals of north-eastern Brazil. Frontiers in Earth Science. 2016;4:29.

[Reference] Alvares CA, Stape JL, Sentelhas P. C, de Moraes G, Leonardo J, & Sparovek G. Köppen's climate classification map for Brazil. Meteorologische Zeitschrift, 2013;22(6): 711-728.

The population of the Northeastern (NE) region of Brazil in 2014 was 56.7 million [3].

Fig C shows the map of Northeastern (NE) region of Brazil. We also highlight (in red) the locations of the six cites where we collected the weather data. The population sizes of the selected six cities were summarised in Table A.

The climate data (temperature and rainfall) used in the modelling was aggregated data from six

Table A: The demographic information of the selected six cities in NE Brazil.

City	State	Population	Region
Fortaleza	Ceara	2,643,247	NE Brazil
Maceio	Alagoas	990,000	NE Brazil
Petrolina	Pernambuco	343,865	NE Brazil
Recife	Pernambuco	1,633,697	NE Brazil
Salvador	Bahia	2,677,000	NE Brazil
Vitória da Conquista	Bahia	260,300	NE Brazil

major cities (Fortaleza of Ceara, Maceio of Alagoas, Petrolina and Recife of Pernambuco, Salvador and Vitória da Conquista of Bahia) all with large populations in NE Brazil. Although spatial differences exist, the temporal patterns of the temperature data are highly correlated in space.

As further confirmation of the uniformity we have correlated temperature time-series (given in Fig D) of all pairs of the six cities studied in our paper, and the average correlation is r = 0.62. Taking all these factors into account, it seems reasonable to spatially aggregate temperature time series and work with the spatial average. For the same reasons, this appears to be reasonable also for rainfall time series.

The daily mean temperature and total rainfall (starting from December 1, 2014) data of the six NE cities were obtained from the online source: https://www.worldweatheronline.com/, and shown in Fig D.

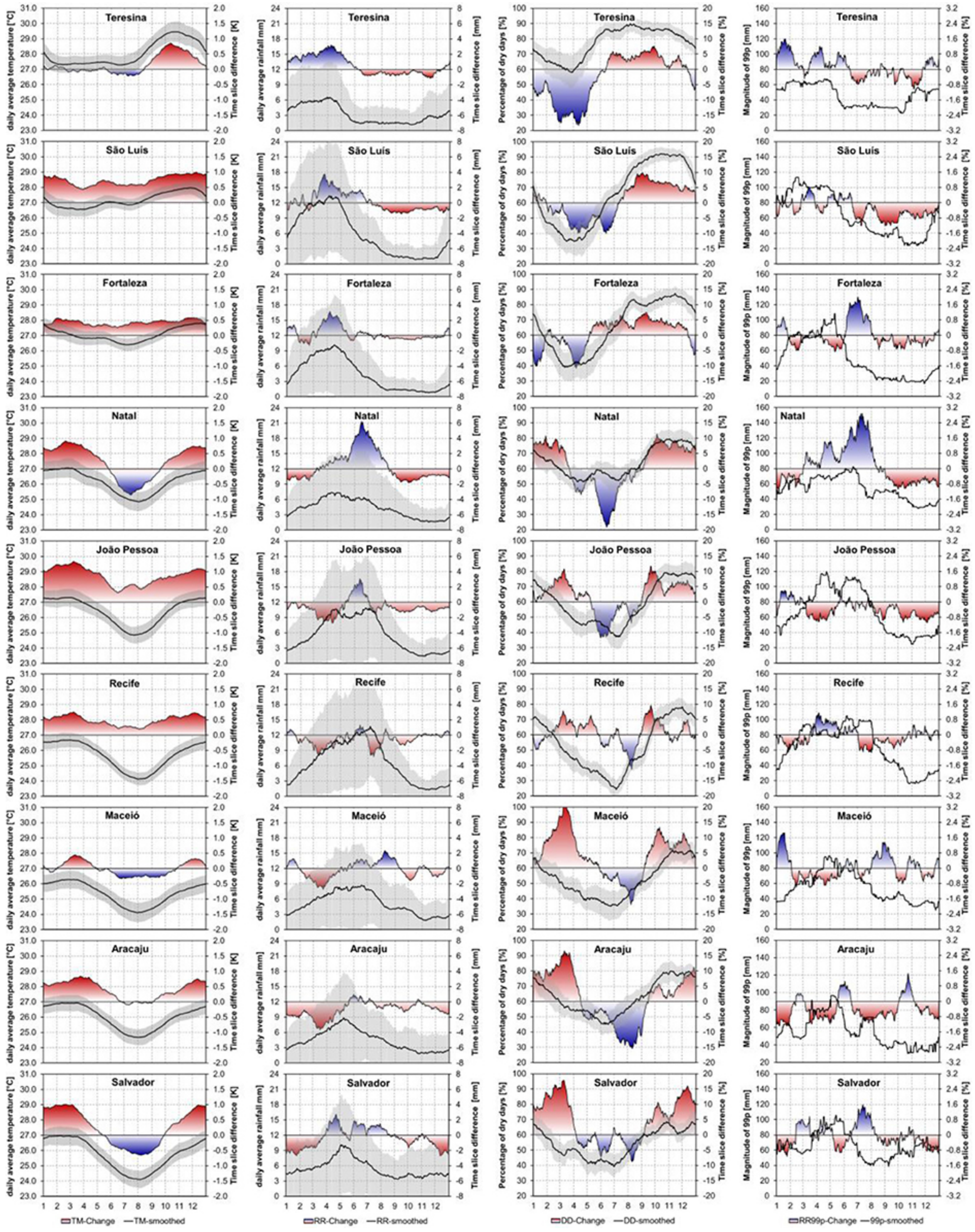

Figure A: Seasonality in daily average temperature (TM, first column), precipitation (RR, second column), dry day (DD, third column), and heavy precipitation (99th percentile, fourth column) frequency at nine stations in NEB within 1961–1990 (black curve and gray area) as well as change (red/blue) in the seasonal cycle in 1991–2011 as compared to 1961–1990. Figure 5 of Hänsel et al., 2016.

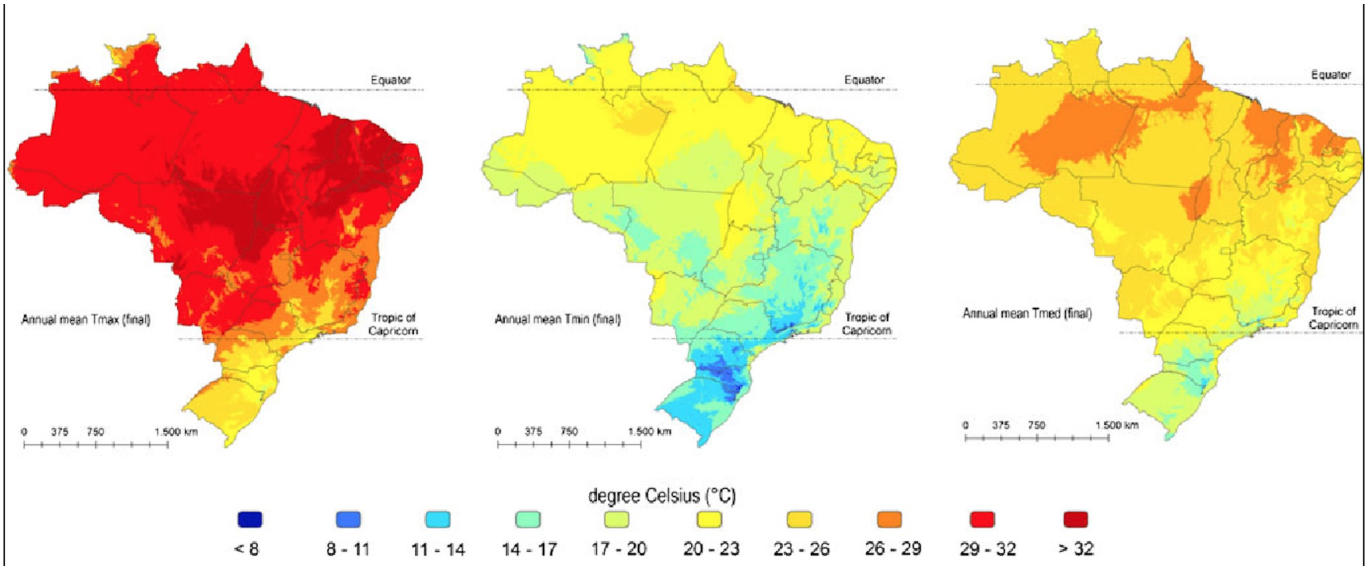

Figure B: Annual maximum, minimum, and mean air temperature maps for Brazil. Figure 8 of Alvares et al., 2012.

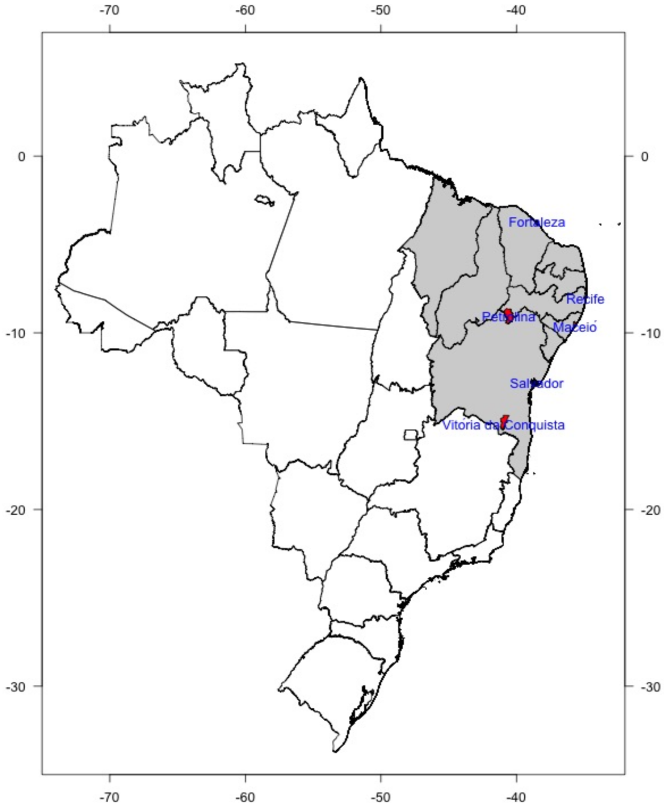

Figure C: The map of Brazil. The Northeastern (NE) region is shaded in grey. The locations of the six cities where we collected weather data are highlighted in red. The horizontal and vertical axes are the longitude and latitude respectively.

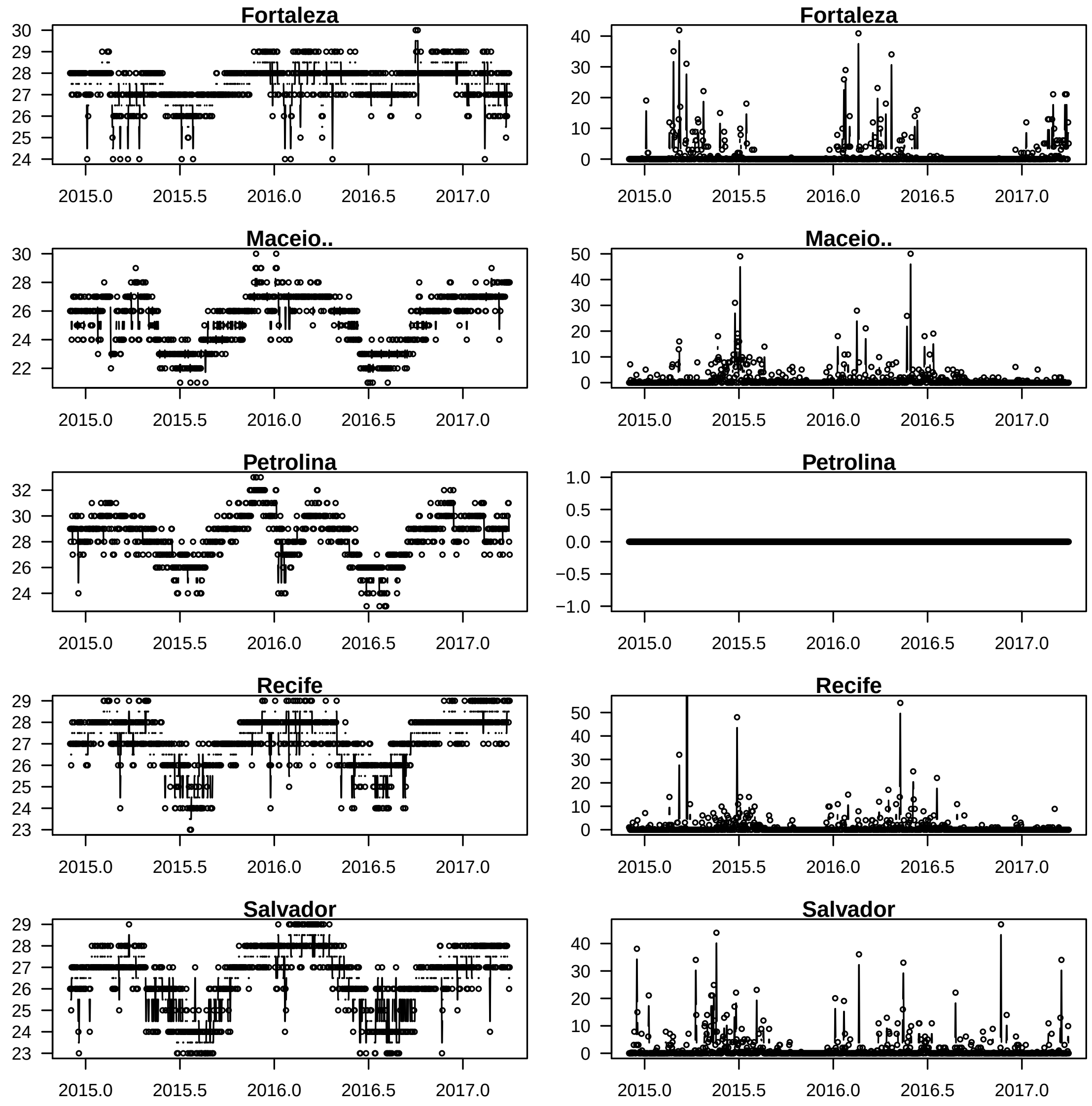

Figure D: Weather data from the selected six Brazilian cities. Left panels: daily mean temperature (°C). Right panels: daily total rainfall (mm).

S1.3 Uncertainty in reproduction number

$\mathcal{R}_0$(t) is not a controllable parameter in our model. Following a previous study [8], to find its 95% confidence interval (CI), we perturb the parameters of p_1, p_2 and p_4 by multiplying log-normally distributed noises and obtain a log-likelihood profile of $\mathcal{R}_0(t)$. We show all estimated $\mathcal{R}_0(t)$ series with a log-likelihood greater than MLL$-0.5 \cdot \chi^2(\text{prob.} \ = 0.95, \text{df} = 1)$ in Fig 4 of the main text (see the green lines), where MLL is the maximum log-likelihood (MLL) of the full model.

S1.4 Fitting Results with Cubic Spline Reconstruction (i.e., no climate variables involved)

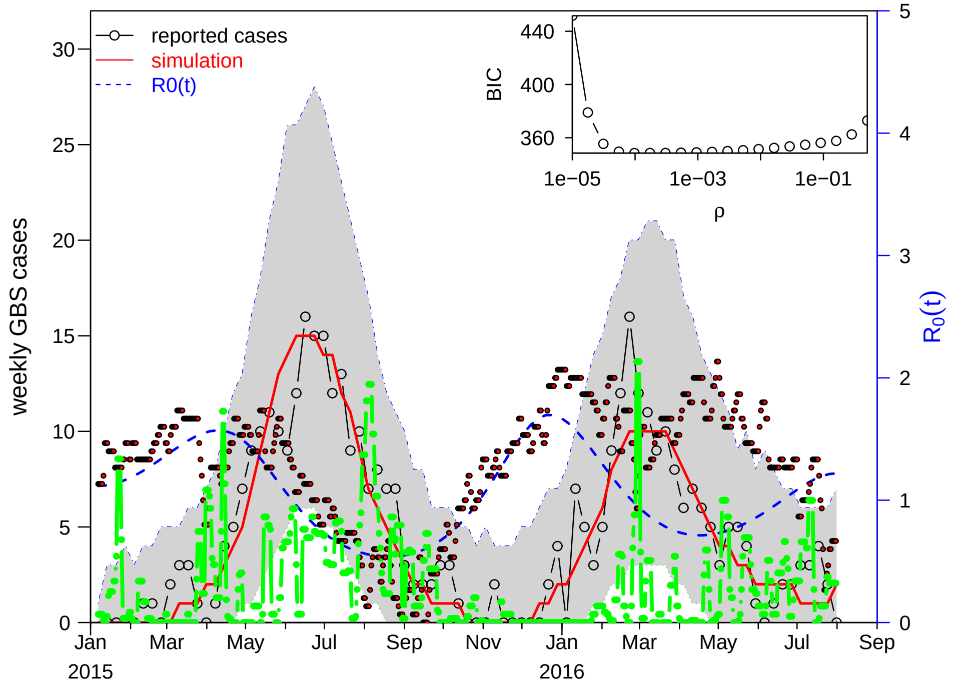

Figure E: Fitting results with a non-mechanistic model, i.e., by cubic spline reconstruction. The black circles are the reported number of GBS cases. The red line is the fitted GBS cases from the simulation and the shaded region represents the 95% CI. The weather data are also shown in this figure. The brown dots are mean daily temperature (in °C) shifted by +1 month and multiplied by 0.4. The green line is mean daily rainfall (in mm) shifted by +1 month and multiplied by 0.4. The inserted panel shows the profile likelihood-based estimation of the parameter ρ with different BICs.

As an alternative to driving $\mathcal{R}_0(t)$ with climate variables as in the main text, we also consider a non-mechanistic time-dependent $\mathcal{R}_0(t)$. We achieve the time-dependent $\mathcal{R}_0(t)$ by modelling $m(t)$ as an exponential cubic spline function of time, t, with a number of nodes, n_m, over the study period. The nodes contain a series of time points (t_i) that determine the time windows, and for each t_i, there is a value of $m(t = t_i) = m_i$ associated. Hence, the nodes are denoted by time and the value of m in pairs, i.e., (t_i, m_i) for $i = 1, \dots, n_m$, and thus, we have a total of $(n_m - 1)$ time windows. The time windows are all set to have identical length, and thus the n_m nodes are evenly distributed over the whole time-line (i.e., during our study period). Therefore, since the study period is fixed as the ZIKV epidemic period, once n_m is determined, all t_is are also determined. The values of all m_i's are estimated to further determine the shape of the spline function of $m(t)$ over the study period. We first search n_m over a range from 3 to 12. As in the main text, the Bayesian information criterion (BIC) is used as the measure of fitting performance. A lower BIC indicates better fitting performance.

It was found that $n_m = 10$ yields the lowest BIC. The fitting results are shown in Fig E. This

non-mechanistic model (i.e., based on cubic spline reconstruction) yields a higher BIC than the climate-driven model of the main text. We estimate that BIC $= 348$ for the non-mechanistic model in Fig E, and BIC $= 341$ for the mechanistic model in the results of the main text. The difference between the BICs is 7, which is not substantial but it is nevertheless significant. The comparison of climate data (brown dots and green line) and the reconstructed $\mathcal{R}_0(t)$ is also provided in Fig E. The mean daily temperature "matches" the reconstructed $\mathcal{R}_0(t)$ reasonably well before March 2016 ($\mathcal{R}_0(t)$: the blue dashed line, temperature: brown dots). The relationship between daily rainfall and $\mathcal{R}_0(t)$ is not evident, and thus we only include the effect of temperature in the main results. Here, we reconstructed the $\mathcal{R}_0(t)$ by the cubic spline function, which is non-mechanistic. In the main text, the $\mathcal{R}_0(t)$ is firstly modelled by the effects of rainfall and temperature. Then, we find the effect of rainfall is not significant (with p-value $>$ 0.05). Therefore, we eliminate the rainfall and keep the temperature to further reconstruct the $\mathcal{R}_0(t)$ as its mechanism.

S1.5 Brief Review of the Zika epidemics Infection Attack Rate

Table B summarises the ZIKV IAR estimation results in the literature of the recent 15 years. It is worth mention that the 63.3% IAR of Salvador, Brazil in 2016 were considered as a sero-positivity values. This indicates that the values may not necessarily be an IAR of the ZIKV [13]. The community in [13, 14] in Salvador, Brazil was the hardest hit region in the 2015-2016 ZIKV outbreak.

Table B: Summary table of infection attack rate (IAR) of Zika epidemic in recent 15 years. All analyses in the literature listed here were based on using the ZIKV incidence data.

Place	Period	IAR [95% CI]	Total population	Sample size	Source
Yap island	2007	73% [68-77%]	6892	5005	[9]
Moorea	2013 - 14	51% [47-55%]	16200	7938	[9]
Tahiti	2013 - 14	54% [49-59%]	178100	87269	[9]
New Caledonia	2014	71% [27-98%]	268767	107507	[9]
French Polynesia	2013 - 14	66% [66-73%]	270000		[10]
Brazil	2014 - Feb 2016	16% [13-18%]	206 million	-	[11]
Colombia	2014 - Feb 2016	4% [3-7%]	48.2 million	-	[11]
Mexico	2014 - Feb 2016	1% [0-2%]	125.9 million	-	[11]
Puerto Rico	2014 - Feb 2016	2% [0-7%]	3.5 million	-	[11]
El Salvador	2014 - Feb 2016	1% [0-13%]	6.3 million	-	[11]
Honduras	2014 - Feb 2016	8% [0-28%]	9.0 million	-	[11]
Haiti	2014 - Feb 2016	43% [1-54%]	10.7 million	-	[11]
Venezuela	2014 - Feb 2016	13% [5-19%]	31.2 million	-	[11]
Salvador, Brazil	Jan 2015 - May 2016	63.3% [59.4-66.8%]	2.9 million	633	[12, 13]
Salvador, Brazil	2015	55%-73%	2.9 million	1453	[14]
Dominican Republic	2015 - 16	female: 0.0764%; male: 0.0272%	10.5 million		[15]
Bahia, Brazil	2015 - 16	32.4% [2.5-94.2%]	15.2 million	-	[16]
Colombia	2015 - 16	20.8% [1.1-50.3%]	48.2 million	-	[16]
French Polynesia	2015 - 16	78.0% [63.5-86.3%]	278260	-	[16]
Feira de Santana, Brazil	2015 - 16	75% [76.9-84.3%]	566000	-	[17]
Wynwood, Florida, Americas	Mar - Aug 2016	14.4% [5.6–27.4%]	17923	-	[18]

S1.6 Code of the model equations and simulation scheme

Please see the attached files.

References

[1] de Oliveira WK, Carmo EH, Henriques CM, Coelho G, Vazquez E, Cortez-Escalante J, Molina J, Aldighieri S, Espinal MA, Dye C. Zika Virus Infection and Associated Neurologic Disorders in Brazil. New England Journal of Medicine. 2017;376(16):1591-3.

[2] Campos GS, Bandeira AC, Sardi SI. Zika virus outbreak, Bahia, Brazil. Emerging infectious diseases. 2015;21(10):1885.

[3] The population statistics in Brazil, the City Population website. http://www.citypopulation.de/php/brazil-metro.php; accessed on May 2017.

[4] The health-related fact sheets of Brazil from the Pan American Health Organization official website. https://www.paho.org/salud-en-las-americas-2017/?p=4246; accessed on September 2019.

[5] Hansel S, Medeiros DM, Matschullat J, Petta RA, de Mendonça Silva I. Assessing homogeneity and climate variability of temperature and precipitation series in the capitals of north-eastern Brazil. Frontiers in Earth Science. 2016;4:29.

[6] Alvares CA, Stape JL, Sentelhas PC, de Moraes Gonçalves JL. Modeling monthly mean air temperature for Brazil. Theoretical and Applied Climatology. 2013;113(3-4):407-27.

[7] Musa SS, Zhao S, Chan HS, Jin Z, He DH. A mathematical model to study the 2014-2015 large-scale dengue epidemics in Kaohsiung and Tainan cities in Taiwan, China. Mathematical biosciences and engineering. 2019;16(5):3841-63.

[8] King AA, Ionides EL, Pascual M, Bouma MJ. Inapparent infections and cholera dynamics. Nature. 2008; 454(7206), 877.

[9] Champagne C, Salthouse DG, Paul R, Cao-Lormeau VM, Roche B, Cazelles B. Structure in the variability of the basic reproductive number (R0) for Zika epidemics in the Pacific islands. Elife. 2016;5:e19874.

[10] Johansson MA, Mier-y-Teran-Romero L, Reefhuis J, Gilboa SM, Hills SL. Zika and the risk of microcephaly. New England Journal of Medicine. 2016;375(1):1-4.

[11] Zhang Q, Sun K, Chinazzi M, Piontt APY, Dean NE, *et al.* Spread of Zika virus in the Americas. PNAS. 2017;E4334-E4343. http://www.pnas.org/cgi/doi/10.1073/pnas.1620161114.

[12] Hay JA, Nouvellet P, Donnelly CA, Riley S (2018) Potential inconsistencies in Zika surveillance data and our understanding of risk during pregnancy. PLoS Negl Trop Dis 12(12): e0006991. https://doi.org/10.1371/journal.pntd.0006991.

[13] Netto EM, Moreira-Soto A, Pedroso C, Höser C, Funk S, Kucharski AJ. High Zika Virus Seroprevalence in Salvador, Northeastern Brazil Limits the Potential for Further Outbreaks. American Soceity for Microbiology. 2017;8(6):1-17. DOI:10.1128/mBio.01390-17.

[14] Rodriguez-Barraquer I, Costa F, Nascimento EJM, Júnior NN, Castanha PMS, et al. Impact of preexisting dengue immunity on Zika virus emergence in a dengue endemic region. Science. 2019;363(6427),:607-610. DOI:10.1126/science.aav6618.

[15] Bowman LR, Rocklöv J, Kroeger A, Olliaro P, Skewes R. A comparison of Zika and dengue outbreaks using national surveillance data in the Dominican Republic. PLoS Negl Trop Dis. 2018;12(11): e0006876. https://doi.org/10.1371/journal.pntd.0006876.

[16] He D, Gao D, Lou Y, Zhao S, Ruan S. A comparison study of Zika virus outbreaks in French Polynesia, Colombia and the State of Bahia in Brazil. Scientific Reports. 2017;7:273. DOI:10.1038/s41598-017-00253-1.

[17] Lourenco J, de Lima MM, Faria NR, Walker A, Kraemer MUG, *et al.* Epidemiological and ecological determinants of Zika virus transmission in an urban setting. eLife. 2017;6:e29820. https://doi.org/10.7554/eLife.29820.001.

[18] Marini G, Guzzetta G, Rosa R, Merler S. First outbreak of Zika virus in the continental United States: a modelling analysis. Eurosurveillance. 2017;22(37). http://dx.doi.org/10.2807/1560-7917.ES.2017.22.37.30612.
